# Supplementary material for: Alternative PCR-Based Approaches for Generation of Komagataella phaffii Strains
Source: Microorganisms. 2023 Sep 12;11(9):2297. doi: 10.3390/microorganisms11092297 (PMC10536657; doi:10.3390/microorganisms11092297)
Supplement: Supplementary file 1 [file microorganisms-11-02297-s001.zip › Supplementary 1. Construction of plasmids.pdf]

1) Construction of pPICZ-PHO5 plasmid

Generation of pPIC9-PHO5 vector was described previously [46, 47]. In brief: *Saccharomyces cerevisiae PHO5* acid phosphatase gene (YBR093C) was amplified using PHO5-F (5'-CGGGATCCCGAGATTACCAA-3') and PHO5-R (5'-CGGAATTCCAAAACTATTGT-3') primers. These primers contain *BamHI* and *EcoRI* sites which were used for cloning the fragment into pPIC9 plasmid (Thermo Fisher Scientific, USA).

To generate pPICZ-PHO5 plasmid in this study the insert fragment was cut out of the pPIC9-PHO5 using *SacI* and *AgeI* restriction enzymes. pPICZα B (Thermo Fisher Scientific, USA) vector was cut using same *SacI* and *AgeI* restriction enzymes and dephosphorylated using FastAP. Vector and insert fragments were separated using agarose gel electrophoresis, purified from gel and joined in ligation reaction. After transformation *E. coli* cells were selected on LB medium with Zeocin™. Resulting plasmids were propagated and extracted from transformants. Map of obtained pPICZ- PHO5 plasmid is presented in Figure S1.

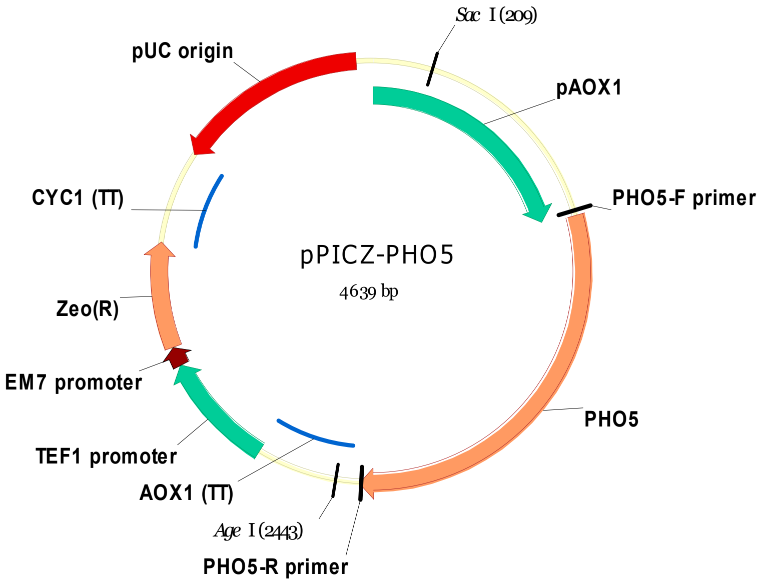

Figure S1. pPICZ-PHO5 plasmid map. Plasmid contains *AOX1* promoter sequence (p*AOX1*), coding sequence of *S. cerevisiae PHO5* acid phosphatase gene (*PHO5*), Zeocin™ resistance gene (*ZeoR*) regulated by prokaryotic (*EM7*) and eukaryotic (*TEF1*) promoters, *CYC1* terminator (*CYC1* (TT)) for *ZeoR* gene and *AOX1* terminator (*AOX1* (TT)) for *PHO5* gene, pUC origin of replication. Green arrows indicate eukaryotic promoters, brown arrow – prokaryotic promoter, orange arrows – coding sequences, blue lines – transcription terminator sequences, red arrow – origin of replication.

Structure of obtained pPICZ-PHO5 plasmid was analyzed using restriction and PCR (Figure S2).

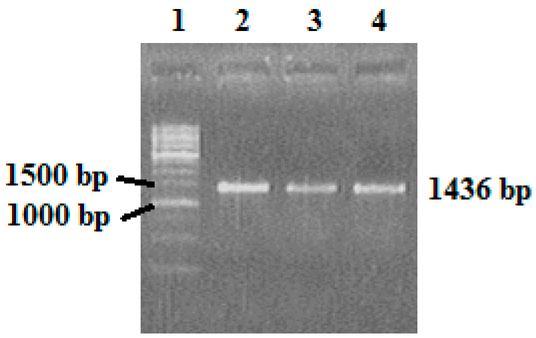

Figure S2. Electropherogram of the results of PCR with plasmids pPIC9-PHO5, pPICZ-PHO5 and pPICK-PHO5 using primers PHO5-F and PHO5-R. Lane 1 – 1 kb DNA Ladder (Evrogen, Russia), lane 2 – PCR amplification of *PHO5* gene with pPIC9-PHO5 as a template (fragment size 1436 bp), lane 3 – PCR amplification of *PHO5* gene with pPICZ-PHO5 as a template (fragment size 1436 bp), lane 4 – PCR amplification of *PHO5* gene with pPICK-PHO5 as a template (fragment size 1436 bp).

2) Construction of pPICZ-eGFP plasmid

pPIC9-eGFP vector was obtained previously in our laboratory. In brief: enhanced green fluorescent protein (*eGFP*) coding sequence was amplified using eGFP-F (5'-ATTACAGGATCCATGGTGAGCAAGGGCG-3') and eGFP-R (5'-ATTACAGAATTCTTACTTGTACAGCTCGTCCATGC-3') primers and pCMV-GFP plasmid [48] as a template. These primers contain *BamHI* and *EcoRI* sites which were used for cloning the fragment into pPIC9 plasmid (Thermo Fisher Scientific, USA).

To generate pPICZ-eGFP plasmid in this study the insert fragment was cut out of the pPIC9-eGFP using *SacI* and *AgeI* restriction enzymes. pPICZα B (Thermo Fisher Scientific, USA) vector was cut using same *SacI* and *AgeI* restriction enzymes and dephosphorylated using FastAP. Vector and insert fragments were separated using agarose gel electrophoresis, purified from gel and joined in ligation reaction. After transformation *E. coli* cells were selected on LB medium with Zeocin™. Resulting plasmids were propagated and extracted from transformants. Map of obtained pPICZ-eGFP plasmid is presented in Figure S3.

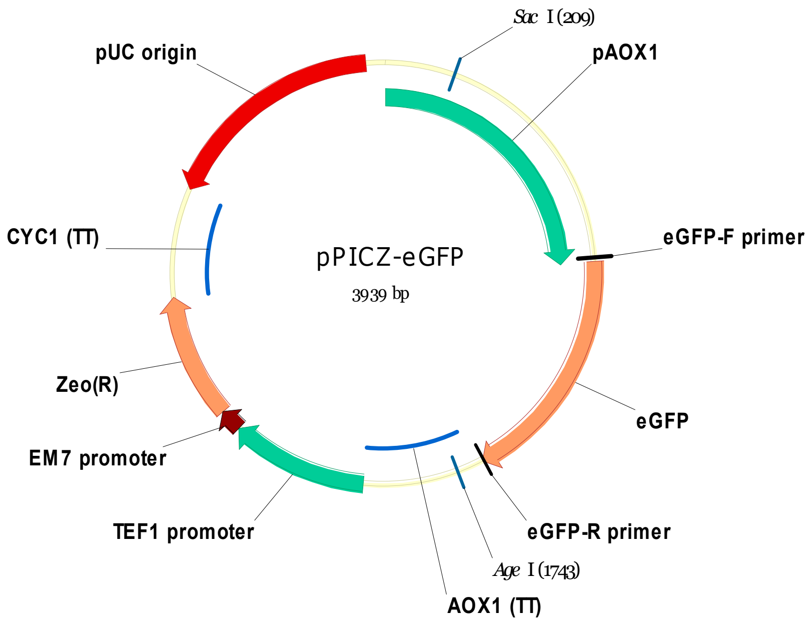

Figure S3. pPICZ-eGFP plasmid map. Plasmid contains *AOX1* promoter sequence (p*AOX1*), coding sequence of *eGFP* gene (*eGFP*), Zeocin^{TM} resistance gene (*ZeoR*) regulated by prokaryotic (*EM7*) and eukaryotic (*TEF1*) promoters, *CYC1* terminator (*CYC1* (TT)) for *ZeoR* gene and *AOX1* terminator (*AOX1* (TT)) for *eGFP* gene, pUC origin of replication. Green arrows indicate eukaryotic promoters, brown arrow – prokaryotic promoter, orange arrows – coding sequences, blue lines – transcription terminator sequences, red arrow – origin of replication.

Structure of obtained pPICZ-eGFP plasmid was analyzed using restriction and PCR (Figure S4).

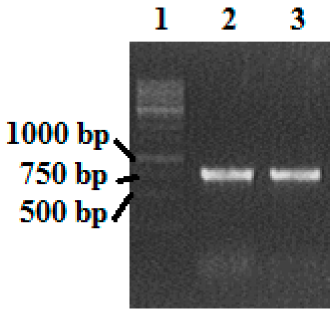

Figure S4. Electropherogram of the results of PCR with plasmids pPIC9-eGFP and pPICZ-eGFP using primers eGFP-F and eGFP-R. Lane 1 – 1 kb DNA Ladder (Evrogen, Russia), lane 2 – PCR amplification of *eGFP* gene with pPIC9-eGFP as a template (fragment size 744 bp), lane 3 – PCR amplification of *eGFP* gene with pPICZ-eGFP as a template (fragment size 744 bp).

3) Construction of pPICK-PHO5 plasmid

Same procedure as for pPICK-Neo plasmid was used to generate pPICK-PHO5 plasmid (see section 6).

After separation by electrophoresis resulting fragments were extracted from agarose gel. 10 µl of mix containing vector and insert (KanR gene) fragments was used for bacterial transformation. Screening of transformants was performed using colony PCR with PHO5-F and PHO5-R primers. Resulting plasmids were propagated and extracted from transformants. Map of obtained pPICZ-PHO5 plasmid is presented in Figure S5.

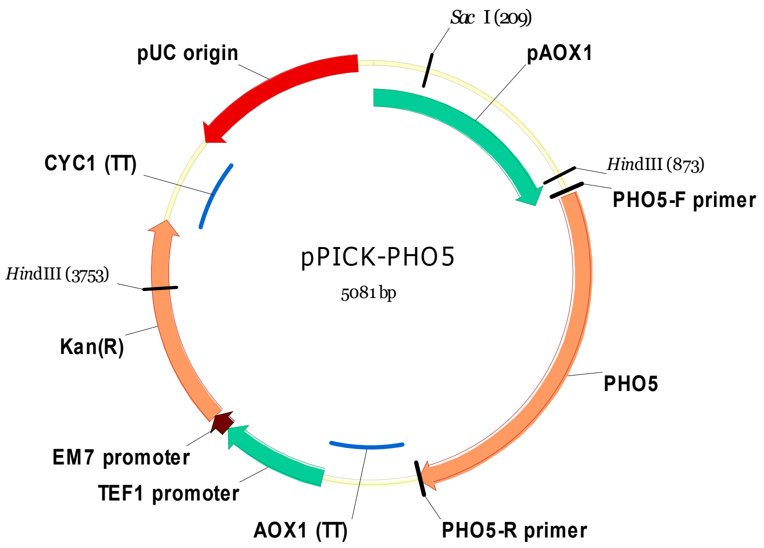

Figure S5. pPICK-PHO5 plasmid map. Plasmid contains *AOX1* promoter sequence (p*AOX1*), coding sequence of *S. cerevisiae PHO5* acid phosphatase gene (*PHO5*), G418 and kanamycin resistance gene (*KanR*) under control of prokaryotic (*EM7*) and eukaryotic (*TEF1*) promoters, *CYC1* terminator (*CYC1* (TT)) for *KanR* gene and *AOX1* terminator (*AOX1* (TT)) for *PHO5* gene, pUC origin of replication. Green arrows indicate eukaryotic promoters, brown arrow – prokaryotic promoter, orange arrows – coding sequences, blue lines – transcription terminator sequences, red arrow – origin of replication.

Structure of obtained pPICZ-PHO5 plasmid was analyzed using restriction and PCR analysis (Figure S6).

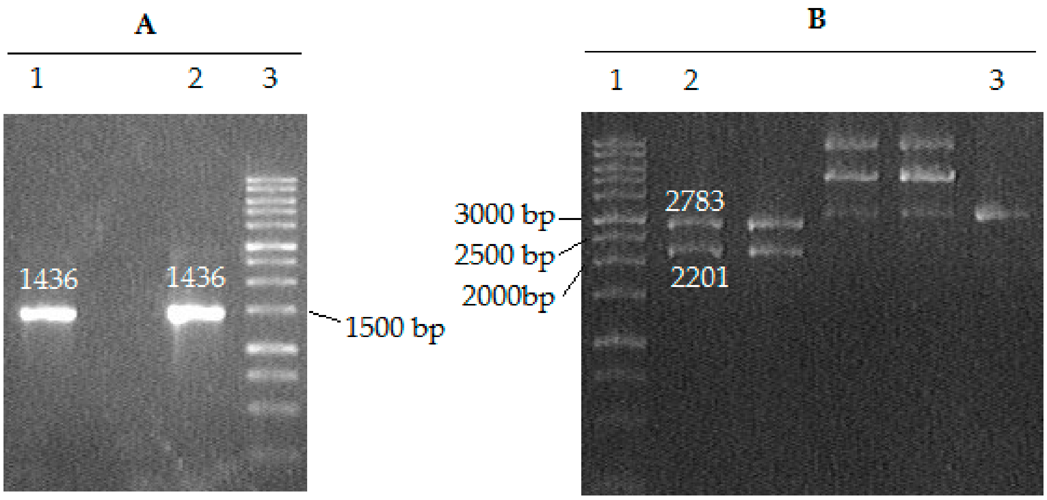

Figure S6. Electropherogram of the results of A) PCR and B) restriction analysis of pPICK-PHO5 plasmid. A) Lane 1 - PCR amplification with PHO5-F/PHO5-R primers and pPICK-PHO5 plasmid as a template (fragment 1436 bp), lane 2 - PCR amplification with PHO5-F/PHO5-R primers and initial pPICZ-PHO5 plasmid as a template (fragment 1436 bp), lane 3 – 1 kb DNA Ladder (Evrogen, Russia). B) Lane 1 - 1 kb DNA Ladder (Evrogen, Russia), lane 2 - results of pPICK-PHO5 plasmid restriction with *HindIII* (fragments 2783 and 2201 bp), lane 3 – native pPICK-PHO5 plasmid.

4) Construction of pPICZ-Neo plasmid

Neo-2/15 amino acid sequence described in [49] was derived from RCSB Protein Data Bank (https://www.rcsb.org/pdb/explore/remediatedSequence.do?structureId=6DG6):

>6DG6:A|PDBID|CHAIN|SEQUENCE

GSHMPKKKIQLHAEHALYDALMILNIVKTNSPPAEEKLEDYAFNFELILEEIARLFESGDQKDEAEKAKRMKEWMKRIKTTASEDEQEEMANAIITILQSWIFS

Nucleotide sequence encoding this protein was designed and optimized using codon frequency table for *K. phaffii* (http://www.kazusa.or.jp/codon/):

>Neo2/15optimized

GGATCTCACATGCCCAAGAAGAAGATCCAGTTACATGCAGAACATGCTCTATATGACGCTTTGATGATTCTGAACATTGTTAAGACAAATTCACCTCCAGCTGAAGAGAAGTTGGAAGATTACGCCTTTAACTTCGAGTTGATTCTTGAGGAAATAGCTAGATTGTTTGAGTCTGGTGATCAGAAAGACGAAGCAGAGAAAGCCAAAAGAATGAAAGAGTGGATGAAACGTATCAAGACTACTGCTAGTGAAGATGAACAAGAAGAGATGGCTAATGCCATTATCACCATACTTCAAAGCTGGATTTTCTCC

XhoI and XbaI restriction sites were added to ends of the sequence. XhoI that we used for cloning into pPICZα B is located inside of the sequence corresponding to αMF secretion signal. To compensate for that part of αMF secretion signal AAAAGA was added at 5’end. Two nucleotides GC were added before XbaI restriction site to ensure that Neo2/15 coding sequence will be in frame with c-myc epitope and 6xHis-tag:

>Neo2/15synthesis

CTCGAGAAAAGAGGATCTCACATGCCCAAGAAGAAGATCCAGTTACATGCAGAACATGCTC
TATATGACGCTTTGATGATTCTGAACATTGTTAAGACAAATTCACCTCCAGCTGAAGAGAAGTTGG
AAGATTACGCCTTTAACTTCGAGTTGATTCTTGAGGAAATAGCTAGATTGTTTGAGTCTGGTGATC
AGAAAGACGAAGCAGAGAAAGCCAAAAGAATGAAAGAGTGGATGAAACGTATCAAGACTACTGCT
AGTGAAGATGAACAAGAAGAGATGGCTAATGCCATTATCACCATACTTCAAAGCTGGATTTTCTCC
GCTCTAGA

The resulting sequence was sent to Lumiprobe (Russia), where it was synthetized and cloned into pUC57 plasmid. pUC57-Neo and pPICZα B (Thermo Fisher Scientific, USA) plasmids were cut by *XhoI* and *XbaI* restriction enzymes. pPICZα B vector was also dephosphorylated using FastAP enzyme. After separation by electrophoresis and extraction from agarose gel, vector (pPICZalpha backbone) and insert (Neo-2/15 sequence) fragments were joined in ligation reaction. After transformation *E. coli* cells were selected on LB medium with Zeocin™. Resulting plasmids were propagated and extracted from transformants. Map of obtained pPICZ-Neo plasmid is presented in figure S7.

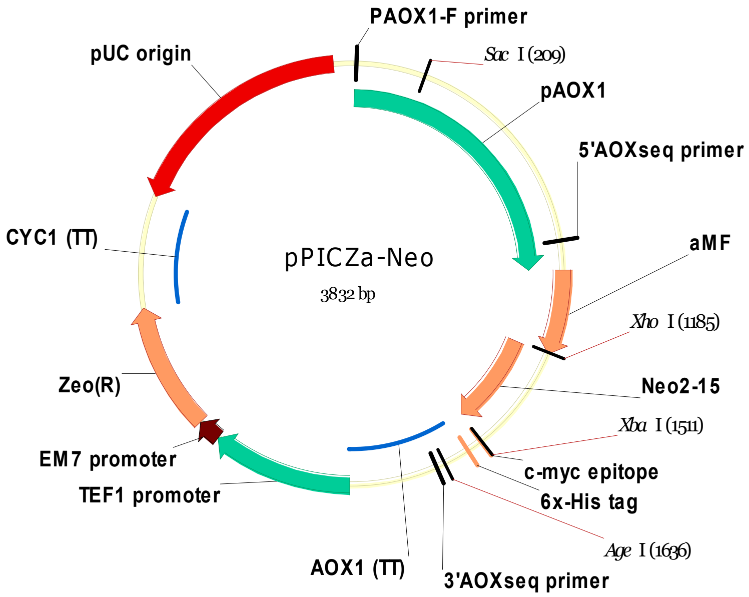

Figure S7. pPICZ-Neo plasmid map. Plasmid contains *AOX1* promoter sequence (p*AOX1*), α-factor sequence (αMF), Neo-2/15 gene sequence optimized for expression in *K. phaffii*, c-myc epitope and 6x-His tag sequence, Zeocin resistance gene (*Zeo(R)*) under control of prokaryotic (*EM7*) and eukaryotic (*TEF1*) promoters, *CYC1* terminator (CYC1 (TT)) for *ZeoR* gene, *AOX1* terminator (*AOX1* (TT)) for *Neo-2/15* gene, and pUC origin of replication. Green arrows indicate eukaryotic promoters, brown arrow – prokaryotic promoter, orange arrows and lines – coding sequences, blue lines – transcription terminator sequences, red arrow – origin of replication.

Structure pPICZ-Neo plasmid of was analyzed using restriction and PCR (Figures S8 and S9).

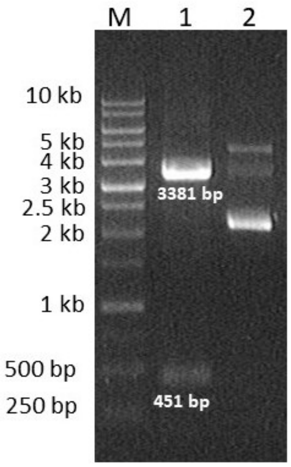

Figure S8. Electropherogram of the results of plasmid pPICZ-Neo restriction analysis. M – 1 kb DNA Ladder (Evrogen, Russia), lane 1 – restriction with *XhoI* and *AgeI* (fragment sizes 3381 bp an 451 bp), lane 2 – native pPICZ-Neo.

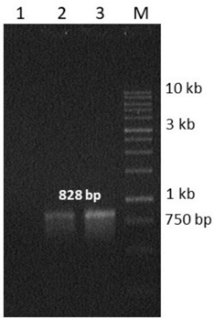

Figure S9. Electropherogram of the results of PCR with plasmids pPICZ-Neo and pPIC9-Neo using primers 5'*AOXseq* (5'-GACTGGTTCCAATTGACAAGC-3') and 3'*AOXseq* (5'-GCAAATGGCATTCTGACATCC-3'). Lane 1 – negative control, lane 2 – PCR with pPICZ-Neo (fragment size 828 bp), lane 3 – PCR with pPIC9-Neo (fragment size 828 bp), M – 1 kb DNA Ladder (Evrogen, Russia).

In resulting plasmid pPICZ-Neo following coding sequence is placed under control of *AOX1* gene promoter:

>codingpPICZ-Neo

ATGAGATTTCCTTCAATTTTTACTGCTGTTTTATTCGCAGCATCCTCCGCATTAGCTGCT
CCAGTCAACACTACAACAGAAGATGAAACGGCACAAATTCCGGCTGAAGCTGTCATCGGTTAC
TCAGATTTAGAAGGGGATTTCGATGTTGCTGTTTTGCCATTTTCCAACAGCACAAATAACGGG
TTATTGTTTATAAATACTACTATTGCCAGCATTGCTGCTAAAGAAGAAGGGGTATCTCTCGAGA
AAAGAGGATCTCACATGCCCAAGAAGAAGATCCAGTTACATGCAGAACATGCTCTATATGACGCTT
TGATGATTCTGAACATTGTTAAGACAAATTCACCTCCAGCTGAAGAGAAGTTGGAAGATTACGCCT
TTAACTTCGAGTTGATTCTTGAGGAAATAGCTAGATTGTTTGAGTCTGGTGATCAGAAAGACGAAG
CAGAGAAAGCCAAAAGAATGAAAGAGTGGATGAAACGTATCAAGACTACTGCTAGTGAAGATGAA
CAAGAAGAGATGGCTAATGCCATTATCACCATACTTCAAAGCTGGATTTTCTCCGC**TCTAGAACAA**
AAACTCATCTCAGAAGAGGATCTGAATAGCGCCGTCGACCATCATCATCATCATCATTGA

Alpha-MF secretion signal

***XhoI* and *XbaI* restriction sites**

Neo-2/15 encoding sequence

Extra nucleotides inserted to keep coding sequence in-frame

c-myc epitope and 6xHis tag sequences

Stop-codon

After synthesis and secretion by *K. phaffii* cells the following protein should be present in the medium:

> Neo2/15secreted

GSHMPKKKIQLHAEHALYDALMILNIVKTNSPPAEEKLEDYAFNFELILEEIARLFESGDQKDEAEKAKR
MKEWMKRIKTTASEDEQEEMANAIITILQSWIFSALEQKLISEEDLNSAVDHHHHHH

It should weight 14.78 kilodaltons according to Protein Molecular Weight calculator (https://www.bioinformatics.org/sms/prot_mw.html).

5) Construction of pPIC9-Neo plasmid

To obtain pPIC9-Neo vector pPICZ-Neo and pPIC9 plasmids were cut with *SacI* and *AgeI* restriction enzymes. pPIC9 vector was also dephosphorylated using FastAP enzyme. After separation by electrophoresis and extraction from agarose gel, vector (pPIC9 backbone) and insert (fragment with Neo-2/15 sequence) were joined in ligation reaction. *E. coli* cells were selected on LB medium with ampicillin. Screening of transformants was performed using colony PCR with PAOX1-F and NeoRQ-R primers. Resulting plasmids were propagated and extracted from transformants. Map of obtained pPICZ-Neo plasmid is presented in figure S10.

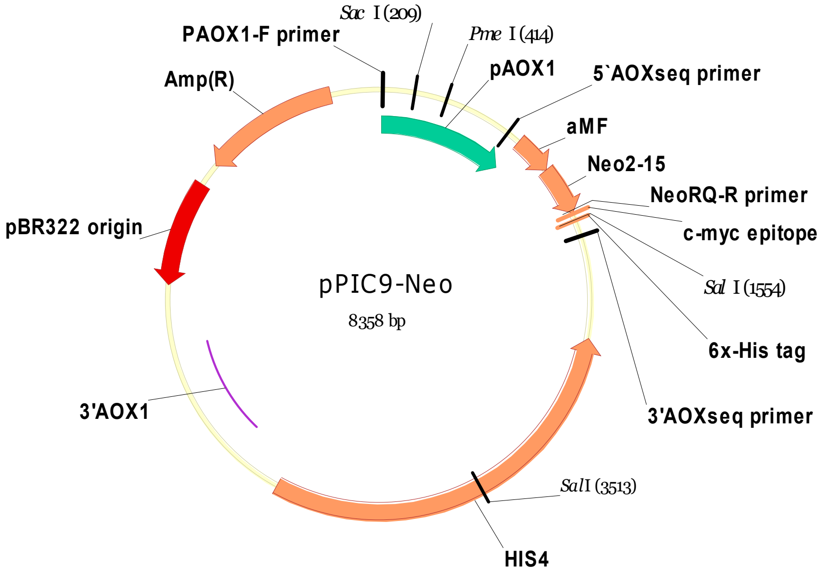

Figure S10. pPIC9-Neo plasmid map. Plasmid contains *AOX1* promoter sequence (*pAOX1*), α-factor sequence (αMF), Neo-2/15 gene sequence optimized for expression in *K. phaffii*, c-myc epitope and 6x-His tag sequence, ampicillin resistance gene (Amp(R)), marker *HIS4* and origin of replication pBR322. Green arrows indicate eukaryotic promoters, orange

arrows and lines – coding sequences, purple line – *AOX1* 3'-fragment for homologous recombination, red arrow – origin of replication.

Structure of obtained pPIC9-Neo plasmid was analyzed using restriction (Figure S11) and PCR (Figure S9).

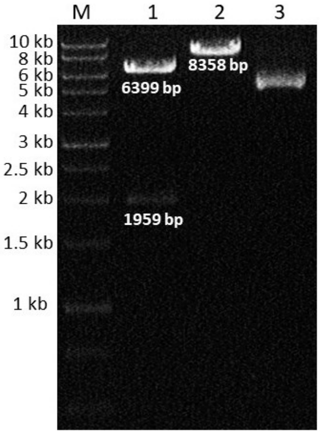

Figure S11. Electropherogram of the results of plasmid pPIC9-Neo restriction analysis. M – 1 kb DNA Ladder (Evrogen, Russia), lane 1 – restriction with *SalI* (fragment sizes 6399 bp and 1959 bp), lane 2 – restriction with *PmeI* (fragment size 8358 bp), lane 3 – native pPIC9-Neo plasmid.

6) Construction of pPICK-Neo plasmid

For iVEC procedure pPICZ-Neo vector backbone without Zeocin™ resistance gene was amplified using iVEC-F and iVEC-R primers. KanR gene from Tn5 encoding an aminoglycoside 3'-phosphotransferase (APH 3' II) was amplified using Kan-F/Kan-R pair of primers and pFA6a-kanMX6 plasmid [50] as a template.

Here for iVEC-F and iVEC-R primers sequences complementary to pPICZ series vectors are marked:

iVEC-F TCGATGAGTTTTTCTAAGGACTGACACGTCCGAC

iVEC-R TTTTCCTTACCCATGGTTTAGTTCCTCACCTTGTC

Here for Kan-F and Kan-R primers sequences complementary to KanR gene in pFA6a-kanMX6 plasmid are marked:

Kan-F GAGGAACTAAACCATGGGTAAGGAAAAGACTCAC

Kan-R CGTGTCAGTCCTTAGAAAAACTCATCGAGCATC

Amplified PCR fragments contain overlapping regions allowing them to be efficiently combined in one plasmid within *E. coli* cell (Figure S12). Here these overlapping regions are marked within the corresponding primers:

iVEC-F	TCGATGAGTTTTTCTAAGGACTGACACGTCCGAC
Kan-R	CGTGTCAGTCCTTAGAAAAACTCATCGAGCATC
iVEC-R	TTTTCCTTACCCATGGTTTAGTTCCTCACCTTGTC
Kan-F	GAGGAACTAAACCATGGGTAAGGAAAAGACTCAC

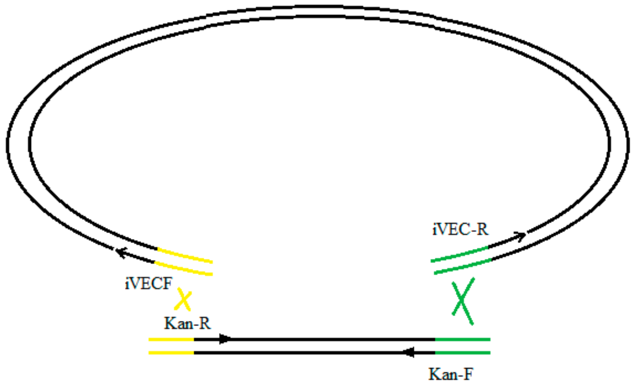

Figure S12. Position of overlapping regions within PCR fragments that are amplified by iVEC-F/iVEC-R and Kan-F/Kan-R pairs of primers.

After separation by electrophoresis resulting fragments were extracted from agarose gel. 10 µl of water solution containing 0,1 pM of vector fragment and 0,3 pM of insert fragment (*KanR* gene) was used for bacterial transformation. Screening of transformants was performed using colony PCR with PAOX1-F and NeoRQ-R primers for the expression cassette. Resulting plasmids were propagated and extracted from transformants. Map of obtained pPICK-Neo plasmid is presented in figure S13.

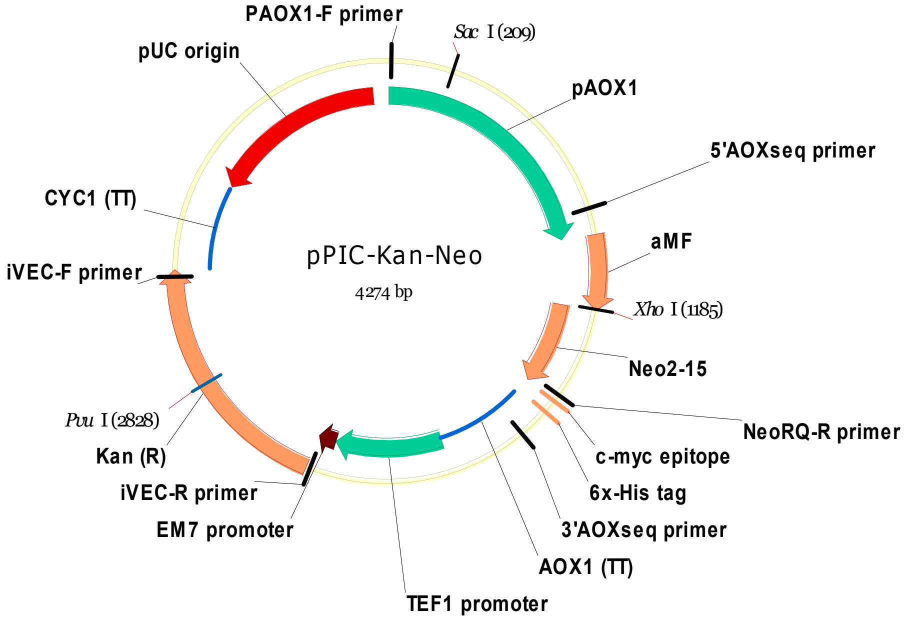

Figure S13. pPICK-Neo plasmid map. Plasmid contains *AOX1* promoter sequence (*pAOX1*), α-factor sequence (αMF), Neo-2/15 gene sequence optimized for expression in *K.*

phaffii, c-myc epitope and 6x-His tag sequence, G418 and kanamycin resistance gene (*KanR*) under control of prokaryotic (*EM7*) and eukaryotic (*TEF1*) promoters, *CYC1* terminator (CYC1 (TT)) for *ZeoR* gene, *AOX1* terminator (*AOX1* (TT)) for *Neo-2/15* gene, and pUC origin of replication. Green arrows indicate eukaryotic promoters, brown arrow – prokaryotic promoter, orange arrows and lines – coding sequences, blue lines – transcription terminator sequences, red arrow – origin of replication.

Structure of obtained pPICK-Neo plasmid was analyzed using restriction (Figure S14) and PCR (Figure S15).

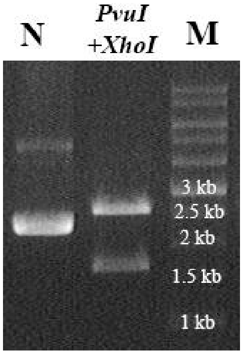

Figure S14. Electropherogram of the results of pPICK-Neo plasmid restriction analysis. Lane N - native pPICK-Neo plasmid, lane *PvuI+XhoI* – results of restriction of pPICK-Neo plasmid with *PvuI* and *XhoI* enzymes (fragments 2631 bp and 1643 bp), lane M – 1 kb DNA Ladder (Evrogen, Russia).

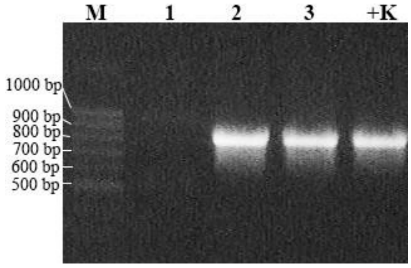

Figure S15. Electropherogram of the results of PCR with pPICK-Neo plasmid. Lane M – 100 bp DNA Ladder (Evrogen, Russia), lane 3 - PCR amplification with 5'*AOXseq* (5'-GACTGGTTCCAATTGACAAGC-3') and 3'*AOXseq* (5'-GCAAATGGCATTCTGACATCC-3') primers and initial pPICK-Neo plasmid as a template (fragment 828 bp), lane +K - PCR amplification with 5'*AOXseq* and 3'*AOXseq* primers and initial pPICZ-Neo plasmid as a template (fragment 828 bp).
