# Supplementary material for: Alternative PCR-Based Approaches for Generation of Komagataella phaffii Strains
Source: Microorganisms. 2023 Sep 12;11(9):2297. doi: 10.3390/microorganisms11092297 (PMC10536657; doi:10.3390/microorganisms11092297)
Supplement: Supplementary file 1 [file microorganisms-11-02297-s001.zip › Supplementary 4. Analysis of K. phaffii transformation using PCR and split-marker approach.pdf]

1)  *K. phaffii* X-33 strain was transformed with two PCR fragments containing *PHO5* and *eGFP* reporter genes and parts of *ZeoR* gene. Three separate transformations were performed. Numbers of resulting colonies are presented in table S1.

Table S1. Numbers of colonies yielded after transformation of X-33 strain with mix of PCR fragments or with single fragments. Results for mean number of colonies and SD were round to integers.

| | Only first PCR fragment (control) | Only second PCR fragment (control) | Mix of two fragments |
|---|---|---|---|
| Replica 1 (number of colonies) | 15 | 33 | 2739 |
| Replica 2 (number of colonies) | 54 | 24 | 1845 |
| Replica 3 (number of colonies) | 79 | 28 | 2176 |
| Mean number of colonies | 49 | 28 | 2253 |
| Standard deviation (SD) | 26 | 4 | 369 |

2)  Transformants were transferred on plates with MM medium and their phenotypes were analyzed. Further we demonstrate the analysis of one such plate as an example (Figures S16-S18 and corresponding Tables S2-S4).

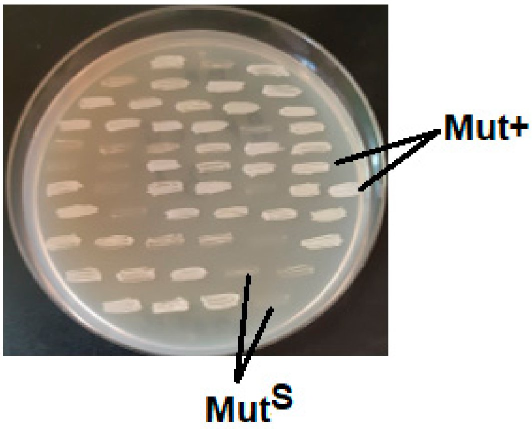


Figure S16. Growth of transformants on MM medium with methanol. Two $Mut^{+}$ and two $Mut^{S}$ colonies are marked as an example of different phenotypes.

Table S2. Results of analysis of methanol utilization by transformants. $Mut^{+}$ are marked as (+), $Mut^{S}$ are marked as (S).

| | 1 | 2 | 3 | 4 | 5 | 6 | 7 |
|---|---|---|---|---|---|---|---|
| 1 | + | S | + | | | | |
| 2 | + | + | + | + | | | |
| 3 | + | + | + | + | + | | |
| 4 | + | + | + | + | S | + | |
| 5 | S | S | S | + | + | + | |
| 6 | S | S | + | + | + | + | |
| 7 | + | S | + | + | S | + | + |
| 8 | + | S | + | + | + | + | |
| 9 | + | + | + | + | S | + | |
| 10 | + | + | + | S | S | | |
| 11 | + | + | + | S | | | |

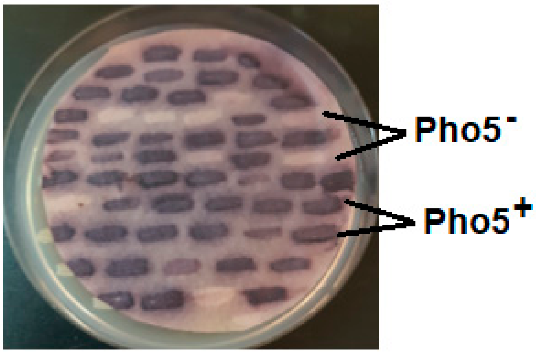


Figure S17. Reporter acid phosphatase activity on the surface of colonies analyzed using qualitative method. Two $Pho5^{+}$ and two $Pho5^{-}$ colonies are marked as an example of different phenotypes.

Table S3. Results of analysis of reporter acid phosphatase activity on the surface of the colonies of transformants. $Pho5^{+}$ are marked as (+), $Pho5^{-}$ are marked as (-). $Mut^{S}$ are transformants are highlighted with red.

| | 1 | 2 | 3 | 4 | 5 | 6 | 7 |
|---|---|---|---|---|---|---|---|
| 1 | + | + | + | | | | |
| 2 | + | + | + | + | | | |
| 3 | + | + | + | - | + | | |
| 4 | + | - | - | - | + | - | |
| 5 | + | + | + | + | + | + | |
| 6 | + | + | + | - | + | - | |
| 7 | + | + | + | + | + | + | + |
| 8 | - | + | + | + | + | + | |
| 9 | + | + | + | + | + | + | |
| 10 | + | + | + | + | + | | |
| 11 | + | + | - | + | | | |

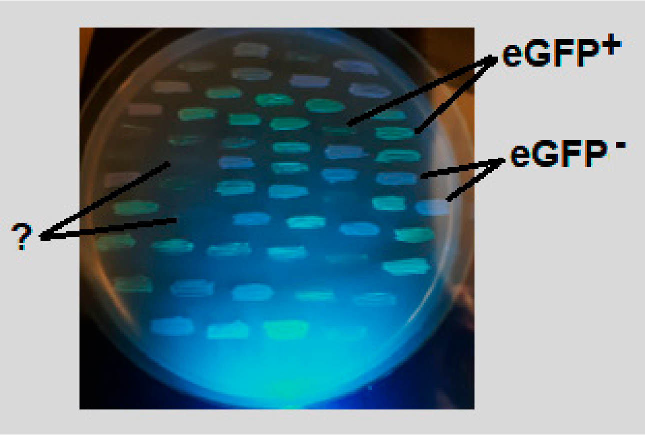


Figure S18. Synthesis of reporter eGFP inside transformant cells visualized using in house equipment. Two eGFP$^+$ (one of them being Mut$^S$ and and another Mut$^+$) and two eGFP$^-$ colonies are marked as an example of different phenotypes. Due to the slow growth of Mut$^S$ strains and low number of cells sometimes it was not easy to determine if eGFP is synthetized by such transformants (marked by ?). In this case additional experiments were performed. Such transformants were grown for 24 hours in 10 ml of BMGY medium with glycerol for biomass growth. Then cells were transferred in 10 ml of BMMY medium with methanol for induction of synthesis of reporter eGFP. Following analysis of fluorescence revealed that all these Mut$^S$ transformants synthetized reporter eGFP.

Table S4. Results of analysis of reporter reporter eGFP synthesis in transformants cells. eGFP$^+$ are marked as (+), eGFP$^-$ are marked as (-). Mut$^S$ are transformants are highlighted with red. Some of them required additional analysis which demonstrated that all of them synthetize eGFP (marked as ?/+).

| | 1 | 2 | 3 | 4 | 5 | 6 | 7 |
|---|---|---|---|---|---|---|---|
| 1 | - | + | - | | | | |
| 2 | - | - | - | - | | | |
| 3 | - | + | + | + | - | | |
| 4 | - | + | + | + | + | + | |
| 5 | + | + | + | + | - | + | |
| 6 | + | ?/+ | - | + | - | - | |
| 7 | - | + | + | - | ?/+ | + | - |
| 8 | + | ?/+ | - | + | - | + | |
| 9 | + | + | + | + | + | + | |
| 10 | + | - | + | + | + | | |
| 11 | - | + | + | + | | | |

3) Phenotypes of at least 90 colonies were analyzed in this way for each transformation (Table S5). For Mut$^+$ colonies all possible phenotypes regarding reporter gene activity were observed (Pho5$^+$/eGFP$^+$, Pho5$^+$/eGFP$^-$, Pho5$^-$/eGFP$^+$, Pho5$^-$/eGFP$^-$). For Mut$^S$ colonies only desired Pho5$^+$/eGFP$^+$ phenotype was observed.

Table S5. Results of phenotype analysis of transformats obtained by PCR and split-marker based approach. Results for mean number of colonies (and SD for it) were round to integers.

| | $Mut^+$ colonies | | $Mut^S$ colonies (Only $Pho5^+$/$eGFP^+$ observed) | |
|---|---|---|---|---|
| | Number | % | Number | % |
| Replica 1 | 92 | 79,3 | 24 | 20,7 |
| Replica 2 | 98 | 81 | 23 | 19 |
| Replica 3 | 108 | 80 | 27 | 20 |
| Mean | 99 | 80,1 | 25 | 19,9 |
| Standard deviation (SD) | 7 | 0,7 | 2 | 0,7 |
