# Supplementary material for: Alternative PCR-Based Approaches for Generation of Komagataella phaffii Strains
Source: Microorganisms. 2023 Sep 12;11(9):2297. doi: 10.3390/microorganisms11092297 (PMC10536657; doi:10.3390/microorganisms11092297)
Supplement: Supplementary file 1 [file microorganisms-11-02297-s001.zip › Supplementary 6. Potential loop-out recombination scenarios in different K. phaffii strains.pdf]

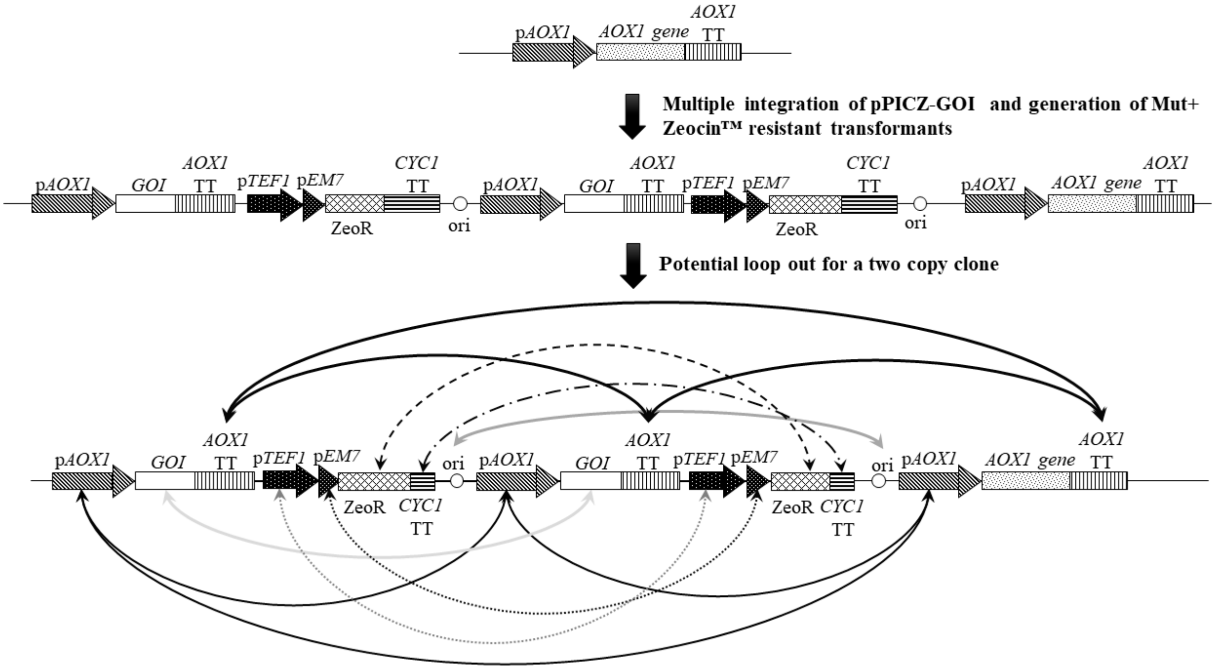

Figure S19. Potential loop out recombination in *K. phaffii* transformants carrying two copies of expression cassette derived through pPICZ transformation and selection in media with high concentration of antibiotic. *AOX1* TT – *AOX1* transcription terminator, *CYC1* TT – *CYC1* transcription terminator, *GOI* – gene of interest, ori – origin of replication, p*AOX1* – *AOX1* promoter, p*EM7* – *EM7* promoter, p*TEF1* – *TEF1* promoter, ZeoR – Zeocin™ resistance gene.

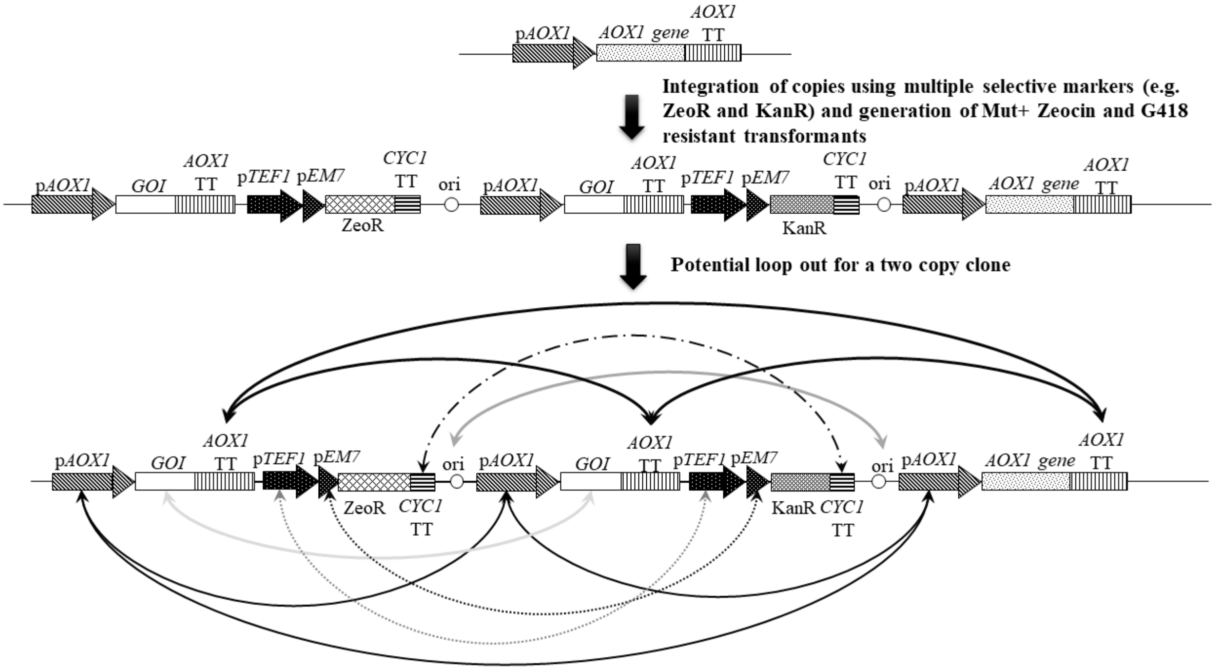

Figure S20. Potential loop out recombination in *K. phaffii* transformants carrying two copies of expression cassette derived using multiple selective markers. *AOX1* TT – *AOX1* transcription terminator, *CYC1* TT – *CYC1* transcription terminator, *GOI* – gene of interest, ori – origin of replication, KanR – G418/kanamycin resistance gene, p*AOX1* – *AOX1* promoter, p*EM7* – *EM7* promoter, p*TEF1* – *TEF1* promoter, ZeoR – Zeocin™ resistance gene.

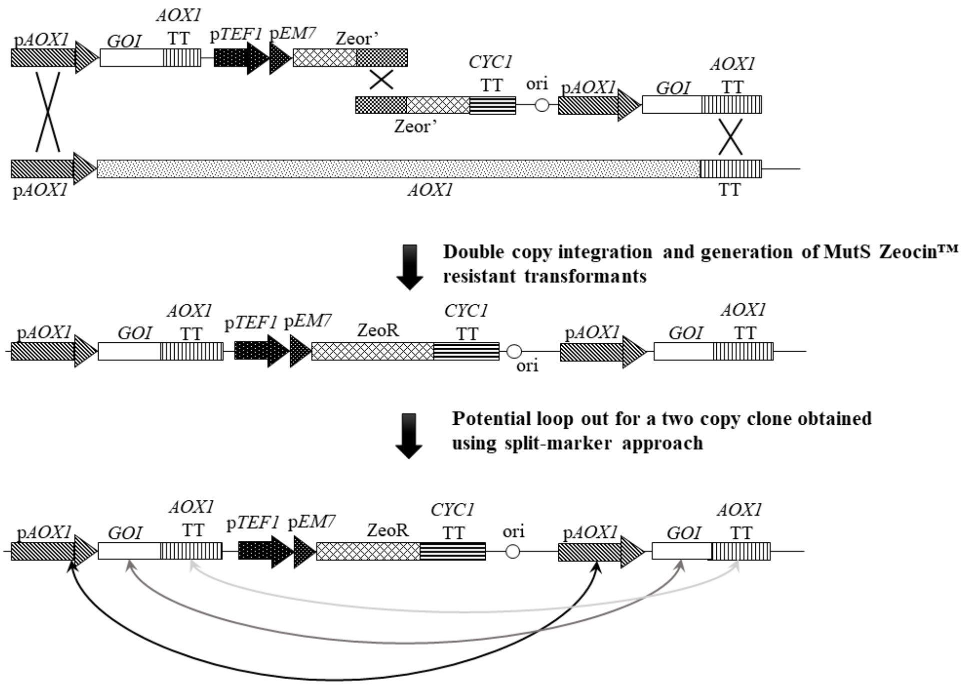

Figure S21. Potential loop out recombination in *K. phaffii* transformants carrying two copies of expression cassette derived using split-marker approach. *AOX1* TT – *AOX1* transcription terminator, *CYC1* TT – *CYC1* transcription terminator, *GOI* – gene of interest, ori – origin of replication, p*AOX1* – *AOX1* promoter, p*EM7* – *EM7* promoter, p*TEF1* – *TEF1* promoter, ZeoR – Zeocin™ resistance gene, Zeor' – truncated Zeocin™ resistance gene.
