# Supplementary material for: Alternative PCR-Based Approaches for Generation of Komagataella phaffii Strains
Source: Microorganisms. 2023 Sep 12;11(9):2297. doi: 10.3390/microorganisms11092297 (PMC10536657; doi:10.3390/microorganisms11092297)
Supplement: Supplementary file 1 [file microorganisms-11-02297-s001.zip › Supplementary 7. The analysis of the stability of the strains with 1-3 copies of Neo-2.15.pdf]

The analysis of the stability of the strains with 1-3 copies of Neo-2/15.

The generation of multicopy *K. phaffii* strains is often associated with the problem of expression cassettes stability. Multiple copies of expression cassettes integrated in *K. phaffii* genome create repeat regions of homology. These regions can be involved in loop out recombination, resulting in expression cassette removal and instability of multicopy strains during prolonged cultivation or storage [33]. One of the strategies to avoid this problem is the use of vectors with different selectable markers. This allows to easily exclude clones that have lost the expression cassette due to recombination by phenotypic analysis of the selectable marker.

We used different cultivation conditions followed by phenotypic analysis to assess the stability of the yeast strains with 1−3 copies of Neo-2/15 expression cassette obtained using multiple selectable markers. In this experiment, we used the following strains: Mut+ N1-GS115 (*P_{AOX1}-Neo2/15 HIS4,* described in detail in the main text of the article) and its derivatives Mut+ N2K-GS115 (*2x P_{AOX1}-Neo2/15 HIS4* ***KanR)*** and N3KZ-GS115 (*3xP_{AOX1}-Neo2/15 HIS4* ***KanR ZeoR***). The derivative strains were generated by serial transformation of N1-GS115 with pPICK-Neo and pPICZ-Neo plasmids.

First, overnight cultures of the strains were inoculated in three replicates in 20 ml of BMGY medium and cultivated for 24 hours at 30 C°. Individual clones were obtained from the cultures by serial dilution and plating on the YEPD medium. After, cells were transferred into 20 ml of BMMY for protein synthesis and cultivated for 72 hours. The supplement with extra 100 µl of methanol was carried out after first 48 hours of cultivation. After 72 hours of incubation, cells were transferred into 20 ml of fresh BMMY and cultivated for an extra 96 hours (the supplement with extra 100 µl of methanol was also carried out after 48 hours). The individual clones were obtained from both BMMY cultures (after 72 and 168 hours of cultivation) as described above, and plated on YEPD medium.

>100 clones from the each sample were plated on master YEPD plates and transferred onto selective media (YEPD+Zeocin™, YEPD+G418, Md) using replica plating. The results of strain growth on the selective media are shown in Table S1. The loss of selectable markers was observed only in the clones carrying three copies of the Neo-2/15 and exposed to continuous methanol induction (72 or 168 hours). The clones that presumably lost a selectable marker were verified by streaking on the corresponding selective media (Figure S19).

Table S6. Results of the analysis of the strains growth in the selective media

Strain	Number of clones analyzed in triplicate	Induction duration	Number of clones that have lost the selectable marker		
			His4	KanR	ZeoR
N1-GS115	184	0 h	0	-	-
	220	72 h	0	-	-
	211	168 h	0	-	-
N2K-GS115	168	0 h	0	0	-
	127	72 h	0	0	-
	101	168 h	0	0	-
N3KZ-GS115	149	0 h	0	0	0
	131	72 h	2	1	0
	162	168 h	1	1	0

«-» the strain initially does not carry the corresponding marker.

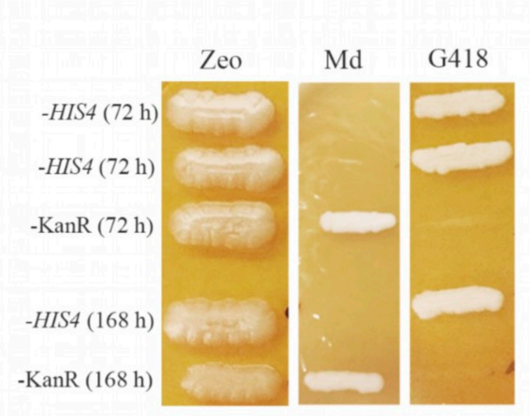

Figure S22. The growth of N3KZ-GS115 (*3x*P_{AOX1}*-Neo2/15 HIS4 KanR ZeoR*) strain clones that have lost selectable markers on YEPD+Zeo, YEPD+G418, and Md. The clones were transferred from the master YEPD plate to the selective media by streaking.

The strain that showed instability carried three copies of the expression cassette in the *AOX1* locus in head-to-tail orientation. The integration of three expression cassettes into *AOX1* locus using pPIC9, pPICZ, and pPICK vectors leads to the formation of 20 regions of homology, increasing the possibility of looping out the expression cassette and their corresponding markers (Figure S20). Notably, we have not observed marker loss in the two-copy strain. The possible reason for this is the use of different vectors (pPICZ and pPIC9) for integration. Naturally, this leads to the formation of only eight homology regions (Figure S21), significantly reducing the chance of recombination.

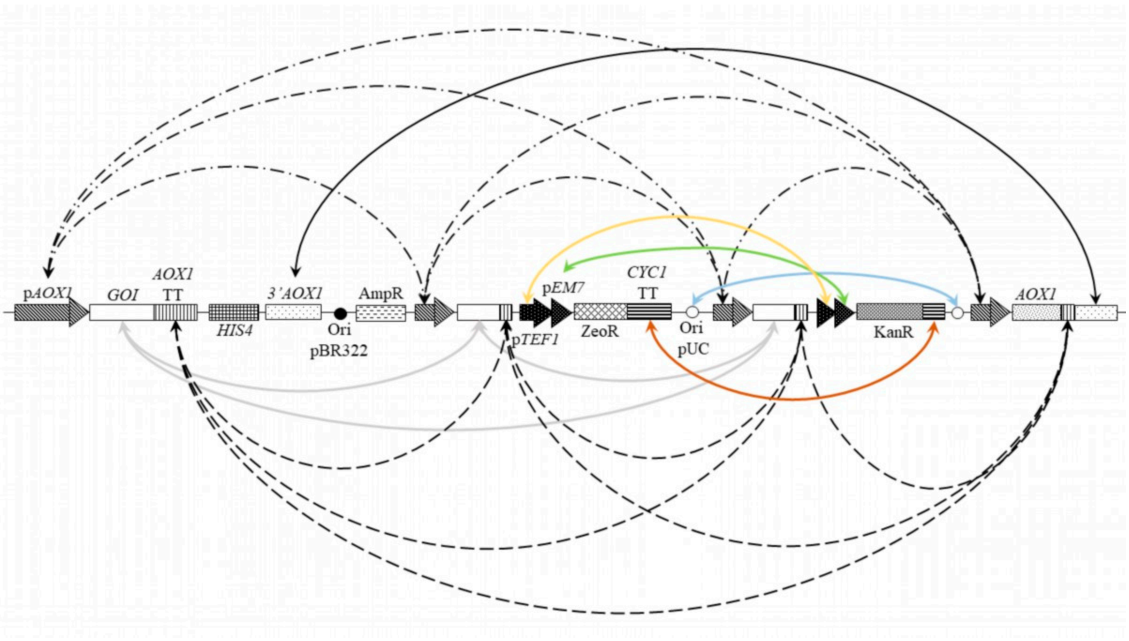

Figure S23. Potential loop out recombination in *K. phaffii* transformants carrying three copies of the expression cassette in *AOX1* locus derived using pPIC9, pPICZ, and pPICK vectors. Note: one of the possible arrangements of the cassettes in the locus is shown. *3'AOX1* – a fragment in the pPIC9 vector homologous to the *3'AOX1* region in the *K. phaffii* genome, *AOX1* TT – *AOX1* transcription terminator, *CYC1* TT – *CYC1* transcription terminator, *GOI* – gene of interest, *HIS4* – *HIS4* marker gene, ori pBR322/pUC – origins of replication, KanR – G418/kanamycin resistance gene, p*AOX1* – *AOX1* promoter, p*EM7* – *EM7* promoter, p*TEF1* – *TEF1* promoter, ZeoR – Zeocin™ resistance gene.

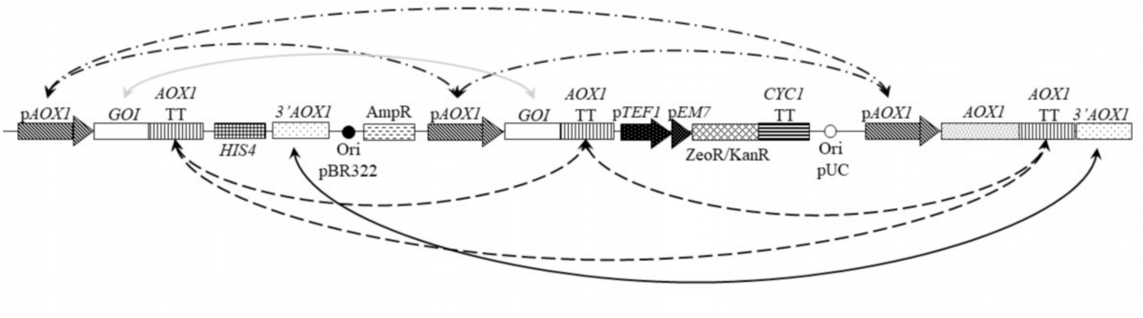

Figure S24. Potential loop out recombination in *K. phaffii* transformants carrying two copies of the expression cassette in *AOX1* locus derived using pPIC9 and pPICZ/pPICK vectors. Note: one of the possible arrangements of the cassettes in the locus is shown. *3'AOX1* – a fragment in the pPIC9 vector homologous to the *3'AOX1* region in the *K. phaffii* genome, *AOX1* TT – *AOX1* transcription terminator, *CYC1* TT – *CYC1* transcription terminator, *GOI* – gene of interest, *HIS4* – *HIS4* marker gene, ori pBR322/pUC – origins of replication, KanR – G418/kanamycin resistance gene, p*AOX1* – *AOX1* promoter, p*EM7* – *EM7* promoter, p*TEF1* – *TEF1* promoter, ZeoR – Zeocin™ resistance gene.

In this experiment we showed that loop outs of selectable markers with expression cassettes are possible in the three-copy Neo-2/15 producing strains. Nevertheless, they can be easily excluded by growth analysis in selective conditions.
